# Sputnik V update: safety and neutralizing antibodies in healthy adults and adolescents

**DOI:** 10.3389/fimmu.2026.1736771

**Published:** 2026-03-26

**Authors:** Inna V. Shuliakova, Daria M. Grousova, Anna A. Iliukhina, Alina S. Dzharullaeva, Nadezhda L. Lubenets, Maria K. Ordzhonikidze, Fatima M. Izhaeva, Alina S. Erokhova, Ilya D. Zorkov, Valentin V. Azizyan, Zoya D. Boeva, Dmitrii A. Reshetnikov, Irina A. Ermolova, Vladislav A. Lega, Anna V. Kovyrshina, Kirill M. Bukhtin, Lyubov P. Romanova, Ekaterina I. Alekseeva, Svetlana N. Borzakova, Svetlana A. Rachina, Marina G. Rusanova, Tatiana A. Ozharovskaia, Olga Popova, Denis I. Zrelkin, Olga V. Zubkova, Amir I. Tukhvatulin, Natalia M. Tukhvatulina, Dmitrii V. Shcheblyakov, Irina A. Favorskaya, Ilias B. Esmagambetov, Andrey P. Karpov, Aleksandr S. Semikhin, Andrey A. Pochtovyi, Boris S. Naroditskiy, Vladimir A. Gushchin, Aleksandr L. Gintsburg, Denis Y. Logunov

**Affiliations:** 1 N.F. Gamalei Federal Research Centre for Epidemiology and Microbiology, Ministry of Health Russian Federation, Moscow, Russia; 2 Moscow State Budgetary Healthcare Institution «Z.A. Bashlyaeva Children's City Clinical Hospital of the Moscow Department of Healthcare», Moscow, Russia; 3 I.M. Sechenov First Moscow State Medical University of the Ministry of Health of the Russian Federation (Sechenov University), Moscow, Russia; 4 Moscow State Budgetary Healthcare Institution "City Polyclinic No. 64 of the Moscow Department of Healthcare", Moscow, Russia; 5 Moscow State Budgetary Healthcare Institution "City Polyclinic No. 62 of the Moscow Department of Healthcare", Moscow, Russia; 6 Department of Medical Genetics and Postgenomic Technologies, I.M. Sechenov First Moscow State Medical University of the Ministry of Health of the Russian Federation (Sechenov University), Moscow, Russia; 7 Department of Virology, Lomonosov Moscow State University, Moscow, Russia

**Keywords:** NtAb, SARS-CoV-2, Sputnik V, vaccine, XBB

## Abstract

**Background:**

During the COVID-19 pandemic, adenoviral vector-based Sputnik V vaccine was used to vaccinate the civilian population of 74 countries worldwide. As part of laboratory monitoring of the effectiveness of the Sputnik V vaccine, a decrease in effectiveness was detected against Omicron BA.5 and XBB variants. XBB variant quickly displaced all previously circulating variants, so the antigen composition of the Sputnik V vaccine was changed to the XBB variant.

**Methods:**

The Gam-COVID-Vac XBB (Sputnik V XBB) vaccine was developed, manufactured, and stored by Gamaleya NRCEM (Moscow, Russia). Two open prospective clinical study of the safety, reactogenicity and immunogenicity of the Sputnik V XBB vaccine was conducted in 50 adult participants over 18 years old and 50 adolescent participants 12–17 years old (ClinicalTrials.gov Identifier: NCT06068569 and NCT06068556). Immunogenicity study included ELISA assay for detection glycoprotein S of the Omicron XBB variant and neutralization assay with viable SARS-CoV-2 virus Omicron ХВВ.1.5, ХВВ.1.9.1, ХВВ.1.16, EG.5.1, ВА.2.86, JN.1, KS.1, XFG.3, NY.2 and PY.2 variants.

**Results:**

Safety profile of the Sputnik V XBB vaccine was consistent with the previous formulation, and no new safety concerns were reported. There were no cases of serious AEs. Seroconversion of antigen-specific IgG on day 42 was 100% in adults and 87.5% in adolescents. We showed robust NtAb response to circulating SARS-CoV-2 variants (ХВВ.1.5, ХВВ.1.9.1, ХВВ.1.16, EG.5.1, ВА.2.86, JN.1, KS.1, XFG.3, NY.2 and PY.2) in vaccinated adults and adolescents, seroconversion rate of NtAb against any circulating variant was 96% in adults and 94% in adolescents.

**Conclusion:**

The results of the clinical trials demonstrated a favorable safety profile and a high level of immunogenicity of Sputnik V XBB in adults and adolescents.

**Clinical Trial Registration:**

ClinicalTrials.gov, identifiers NCT06068569 and NCT06068556.

## 1 Introduction

Coronavirus disease 2019 (COVID-19) is a viral respiratory disease that was first reported in Wuhan (China) in late December 2019 (1). On March 11, 2020, World Health Organization (WHO) declared a pandemic of COVID-19 (2). The causative agent of this disease is severe acute respiratory syndrome coronavirus 2 (SARS-CoV-2), belonging to the *Coronaviridae* family, genus *Betacoronavirus*. According to the WHO, as of October 06, 2025, more than 778 million cases of infection caused by SARS-CoV-2 have been laboratory-confirmed, of which more than 7 million have been fatal. In the Russian Federation, more than 24.9 million cases of the disease have been registered, of which more than 404 thousand have been fatal (3). The most effective way to combat the pandemic is vaccination, which helps limit the spread of infection and reduce the number of COVID-19-associated deaths.

A vaccine for the prevention of COVID-19 based on recombinant human adenoviruses, Gam-COVID-Vac (Sputnik V), was developed at the N.F.Gamaleya National Research Center of Epidemiology and Microbiology (Gamaleya NRCEM, Moscow, Russia) (4, 5). During the pandemic, the Sputnik V vaccine was widely used to vaccinate the civilian population not only in Russia but also abroad (6–17).

The introduction of vaccines into clinical practice in different countries has significantly reduced the incidence and severity of COVID-19 in vaccinated population. The effectiveness of the original (Wuhan variant antigen) Sputnik V vaccine against COVID-19 infection among adults over 18 years of age was more than 90% in phase 3 clinical studies (4). However, the change in the landscape of circulating variants of the virus led to a decrease in the effectiveness of post-vaccination and post-infection immunity, which required the introduction of additional prevention measures - monitoring the effectiveness of the vaccines used and promptly changing the antigen composition. As part of laboratory monitoring of the effectiveness of the Sputnik V vaccine, a decrease in effectiveness was detected against Omicron BA.5 (18) and XBB variants. Considering the significant decrease in effectiveness to XBB variant, as well as the fact that the XBB displaced all previously circulating virus variants, the antigen composition of Sputnik V was updated for the XBB variant. This study presents the results of clinical trials of the Sputnik V vaccine with an updated antigen composition in adults and adolescents.

## 2 Materials and methods

### 2.1 Ethics statement

The studies were reviewed and approved by the appropriate national and local competent authorities, including the Regulator (Department of State Regulation for Medicine Distribution: APPROVAL #509 and #508, 13.09.2023) and Ethics Committee of the Ministry of Health of the Russian Federation. These trials were registered with clinicaltrials.gov (ClinicalTrials.gov Identifier: NCT06068569 and NCT06068556).

### 2.2 Study design and participants

We conducted two open prospective clinical trials of the safety, reactogenicity and immunogenicity of Gam-COVID-Vac XBB vaccine in adults (18+ y.o.) and in adolescents (12–17 y.o.). All participants provided written informed consent before enrollment in the study and were immunized at full dose. Inclusion, non-inclusion and exclusion criteria of the clinical trial in adults (NCT06068569) are presented in **Supplementary Table 1**. Inclusion, non-inclusion and exclusion criteria of the clinical trial in adolescents (NCT06068556) are presented in **Supplementary Table 2**.

### 2.3 Vaccine

The Gam-COVID-Vac XBB (Sputnik V XBB) vaccine consists of a recombinant replication-defective adenovirus type-26 carrying gene of SARS-CoV-2 full-length surface glycoprotein S from SARS-CoV-2 XBB.1 (rAd26-S), and a recombinant replication-defective adenovirus type-5 carrying the same gene (rAd5-S) (18). Both components were developed, manufactured, and stored by Gamaleya NRCEM according to good manufacturing practices. A full dose of the vaccine for adults was 10^11^ viral particles (vp) per dose for both rAds, for adolescents – 2x10^10^ vp per dose for both rAds.

### 2.4 Procedures

Study of the vaccine for adults was carried out at a City Polyclinic No. 64 of the Moscow City Health Department and City Polyclinic No. 62 of the Moscow City Health Department. For clinical trial 51 participants aged 18+ years old were screened. Study of the vaccine for adolescents was carried out at Children’s City Clinical Hospital named after Z.A. Bashlyaeva of the Moscow City Health Department and Sechenov University. For clinical trial 50 participants aged 12–17 years old were screened. All participants signed informed consent. Healthy participants of both sexes, who signed an informed consent form, who had a negative PCR result for SARS-CoV-2 performed on screening, and vaccinated more than 6 months ago. Vaccine was administered intramuscularly into the deltoid muscle (*musculus deltoideus*).

The study included screening (inclusion in the study of participants who met the selection criteria) and the study itself - administration of the two doses of the vaccine at day 1 and day 21 and subsequent observation for 42 days from the administration of the first dose of the vaccine, 90 days - telephone contact. Injection-site reactions, systemic reactogenicity, and medication use to alleviate such symptoms were monitored for 7 days after each vaccine component administration and at days 42/90. Immunogenicity was assessed by several parameters: neutralizing antibodies (NtAb) and antigen-specific IgG before vaccine administration, as well as on days 21 (before administration of the second dose), 28, and 42 of the study. Safety assessment was based on the detection of AEs within 7 days after each administration of the drug and SAEs and AEs of special interest during the entire observation period.

### 2.5 Safety study

During the vaccination period and the entire subsequent observation period, safety information was collected from the participants on the following parameters: local reactions (hyperemia at the injection site, swelling (infiltrate), pain, enlargement of regional lymph nodes, rash, itching) and general reactions (increased body temperature, deterioration of health, headache, dizziness, decreased appetite, insomnia, nausea, vomiting, dyspepsia, weakness, sweating, muscle and joint pain, abdominal pain, convulsions, allergic reactions, anaphylactic reactions, etc.). Local and general reactions were recorded by the physician investigator based on body temperature measurements, examination, and questioning of subjects, conducted in accordance with the visit schedule and study procedures. After administration of the vaccine, the participant was observed for 30–60 min.

### 2.6 Immunogenicity study

#### 2.6.1 Neutralization assay

The used SARS-CoV-2 viruses were obtained from the State Virus Collection of the Gamaleya NRCEM: ХВВ.1.5 - hCoV-19/Russia/MOW-PMVL-OM0223O13/2023; ХВВ.1.9.1 –hCoV-19/Russia/MOW-PMVL-OM0223O11/2023; ХВВ.1.16 - hCoV-19/Russia/MOW-PMVL-OM0223O420/2023; EG.5.1 - hCoV-19/Russia/SPE-RII-21139S/2023; ВА.2.86 – hCoV-19/Russia/SPE-RII-MH147243S/2023; JN.1 - hCoV-19/Russia/MOW-PMVL-LSCV-LD134/2023; KS.1 – hCoV-19/Russia/SPE-RII-MH183935S/2024, XFG.3, NY.2 and PY.2 (data not published in GISAID). Viral isolates were obtained from nasopharyngeal swabs of patients (informed consent was provided) with COVID-19 from Moscow and St. Petersburg (Russia). All studies with viable SARS-CoV-2 virus were conducted in BSL-3 conditions. The production of various variants of the SARS-CoV-2 virus was carried out on the Vero E6 cell culture. The titer of the infectious virus was determined on the Vero E6 cell culture by determining the 50% tissue culture infectious dose (TCID_50_). The TCID_50_ titer was calculated using the Spearman-Kerber method.

The neutralization test was carried out at a constant dose of the virus (100 TCID_50_) with different dilutions of the participants’ blood serum as described earlier (4, 5). Briefly, inactivated sera (56 °C for 30 minutes) were diluted in DMEM culture medium supplemented with 2% inactivated fetal bovine serum, then mixed with 100 TCID_50_ of the SARS-CoV-2 virus, incubated for 1 hour at 37 °C, and added to Vero E6 cells. The cells were then incubated at 37 °C in 5% CO_2_. The results were assessed 96 hours after the reaction. The titer of NtAb of the serum was taken as its highest dilution, at which the cytopathic effect was 100% suppressed in ≥2 of 3 replicate wells.

#### 2.6.2 ELISA

The titer of IgG antibodies specific to the S protein of the SARS-CoV-2 Omicron XBB virus was determined by enzyme-linked immunosorbent assay (ELISA). Competitive ELISA was performed to detect antibodies specific to the vaccine antigen (glycoprotein S of the Omicron XBB variant). The first step was to eliminate antibodies with high specificity to ancestral SARS-CoV-2 variant (antibodies after a previous infection or vaccination) from serum samples: two-fold dilutions of participants’ blood sera were prepared in the “Red Buffer” (S012, Hema, Russia), then mixed with 100 ng of the recombinant glycoprotein S of the SARS-CoV-2 Wuhan variant virus, incubated for 1 hour at 37°C on a shaker (100 rpm). This allowed excluding Wuhan-specific antibodies from subsequent reactions by formation of antibody-protein complexes. Then the sera dilutions were added to 96-well plates with immobilized glycoprotein S of the SARS-CoV-2 XBB, incubated for an hour at 37°C on a shaker (100 rpm), washed with a wash buffer (phosphate-buffered saline with 0.1% Tween 20), a solution of secondary antibodies specific for human IgG labeled with horseradish peroxidase (A8792, Sigma) was added, incubated for an hour at 37°C on a shaker (100 rpm), washed with washing buffer, tetramethylbenzidine (TMB) solution was added, incubated at room temperature for 15 minutes and the reaction was inhibited with 1 M sulfuric acid solution. Optical density (OD) was measured using a Multiskan FC plate photometer (Thermo Scientific) at 450 nm. The highest serum dilution, where the OD value exceeds the OD of the control serum in the same dilution by at least 2 times, was taken as the antibody titer.

### 2.7 Outcomes

The primary objectives of clinical trials were to evaluate safety and immunogenicity of heterologous adenoviral-based COVID-19 vaccine. The primary outcome for safety evaluation was an assessment of the frequency, nature, and severity of adverse events with the use of the study vaccine. The primary outcome for immunogenicity evaluation was NtAb levels at days 1, 21, 28, 42.

The secondary safety outcomes included dynamics of vital signs (blood pressure, heart rate, respiratory rate, body temperature). The secondary immunogenicity outcomes included determination of NtAb seroconversion, IgG level at days 1, 21, 28, 42, IgG seroconversion.

### 2.8 Statistical analysis

The sample size was selected in accordance with the regulatory Decree of the Government of the Russian Federation of 03.04.2020 N 441. The sample size of total 50 was expected to produce reliable data on adverse events and immunogenicity. All statistical calculations were performed in the GraphPad Prism 10. The normality of data distribution was evaluated in the d’Agostino-Pearson test. Comparison of paired samples was performed by the Wilcoxon test. For statistical analysis, if a serum sample had no neutralizing activity against the SARS-CoV-2 virus at the initial dilution (1/5), such samples were assigned a value of 1.25 for statistical calculations.

## 3 Results

Between October 10, 2023 and January 10, 2024, we conducted open prospective clinical study of the safety, reactogenicity and immunogenicity of Sputnik V XBB vaccine after administration to healthy adults of both sexes over 18 years of age. The clinical trial of the safety, reactogenicity and immunogenicity of Sputnik V XBB vaccine for adolescents was conducted between November 01, 2023 and May 31, 2024.

In the study for adults, 51 participants were screened, 50 participants of both sexes aged 20–71 years were included in the study, the average age was 42 ± 12.3 years. In the study for adolescents, 50 participants were screened, all participants of both sexes aged 12–17 years were included in the study, the average age was 14.8 ± 1.7 years. **Table 1** presents the demographic and anthropometric characteristics of the study subjects. All participants received two doses of the Sputnik V XBB vaccine.

**Table 1:** Baseline characteristics of participants.

	Adults (n=50)	Adolescents (n=50)
Sex, Male	37 (74%)	19 (38%)
Average height, m	1.75 ± 0.08	1.66 ± 0.10
Average weight, kg	84.4 ± 16.2	56.9 ± 11.5
Age, years	42 ± 12.3	14.8 ± 1.7
Ethnicity		
White	49 (98%)	50 (100%)
Asian	1 (2%)	0 (0%)
Comorbidities	6 (12%)	6 (12%)
Hypertensive heart disease [10020823]	4 (8%)	0 (0%)
Obesity [10029883]	1 (2%)	0 (0%)
Gout [10018627]	1 (2%)	0 (0%)
Type 2 diabetes mellitus [10067585]	1 (2%)	0 (0%)
Dust allergy [10077439]	1 (2%)	0 (0%)
Aortic valve stenosis [10002918]	1 (2%)	0 (0%)
Oral allergy syndrome [10068355]	0 (0%)	4 (8%)
Allergy to arthropod bite [10058285]	0 (0%)	1 (2%)
Myopia [10028651]	0 (0%)	2 (4%)
Contusion [10050584]	0 (0%)	1 (2%)
Acne infantile [10000507]	0 (0%)	1 (2%)
Menstruation irregular [10027339]	0 (0%)	1 (2%)

Variable indicators are presented as mean and standard deviation. MedDRA number for comorbidities is presented in square brackets.

### 3.1 Safety

During the study, participants were monitored for 90 days after vaccination.

Through the study, systemic and local reactions (**Table 2**) and changes in laboratory parameters were among the adverse events (AEs). The most common systemic and local reactions were mild and presented by pain at the injection site, injection site erythema, influenza like illness and asthenia. Changes in laboratory parameters were mild and transient. All AEs registered in 18+ participants were mild (grade 1), while 4 adolescent participants (8%) had moderate (grade 2) influenza like illness.

**Table 2:** Solicited systemic and local adverse events after vaccination.

	Adults (n=50)		Adolescents (n=50)	
	N	%	N	%
Any AEs	34	68%	34	68%
General disorders and administration site conditions [10018065]	31	62%	33	66%
Injection site pain [10022086]	27	54%	17	34%
Influenza like illness [10022004]	7	14%	9	18%
Asthenia [10003549]	5	10%	–	
Pyrexia [10037660]	4	8%	13	26%
Injection site erythema [10022061]	3	6%	5	10%
Hyperhidrosis [10020642]	2	4%	–	
Chills [10008531]	2	4%	–	
Feeling hot [10016334]	1	2%	–	
Injection site edema [10022085]	–		1	2%
Musculoskeletal and connective tissue disorders [10028395]	8	16%	–	
Musculoskeletal pain [10028391]	4	8%	–	
Myalgia [10028411]	3	6%	–	
Arthralgia [10003239]	1	2%	–	
Gastrointestinal disorders [10017947]	4	8%	–	
Diarrhea [10012735]	3	6%	–	
Abdominal discomfort [10000059]	1	2%	–	
Respiratory, thoracic and mediastinal disorders [10038738]	3	6%	–	
Rhinorrhea [10039101]	1	2%	–	
Cough [10011224]	1	2%	–	
Oropharyngeal pain [10068319]	1	2%	–	
Nasal congestion [10028735]	1	2%	–	
Nervous system disorders [10029205]	2	4%	4	8%
Headache [10019211]	2	4%	3	6%
Dizziness [10013573]	–		1	2%

The table shows the total number and percent of participants with developed AEs. MedDRA number is presented in square brackets.

None of the AEs led to the withdrawal of the participant from the study or the withdrawal of the study drug. In general, the AEs identified during the study were characteral for most vaccines (especially based on recombinant viral vectors). There were no cases of serious AE. In general, safety profile of the Sputnik V XBB vaccine was consistent with the previous (Wuhan variant antigen) formulation of the Sputnik V (4, 5).

### 3.2 Immunogenicity

During the study, the main parameters to assess post-vaccination humoral immunity in participants were: the level of antigen-specific IgG to glycoprotein S of SARS-CoV-2 XBB variant and the level of neutralizing antibodies to different variants of the SARS-CoV-2 virus.

**Figure 1:**
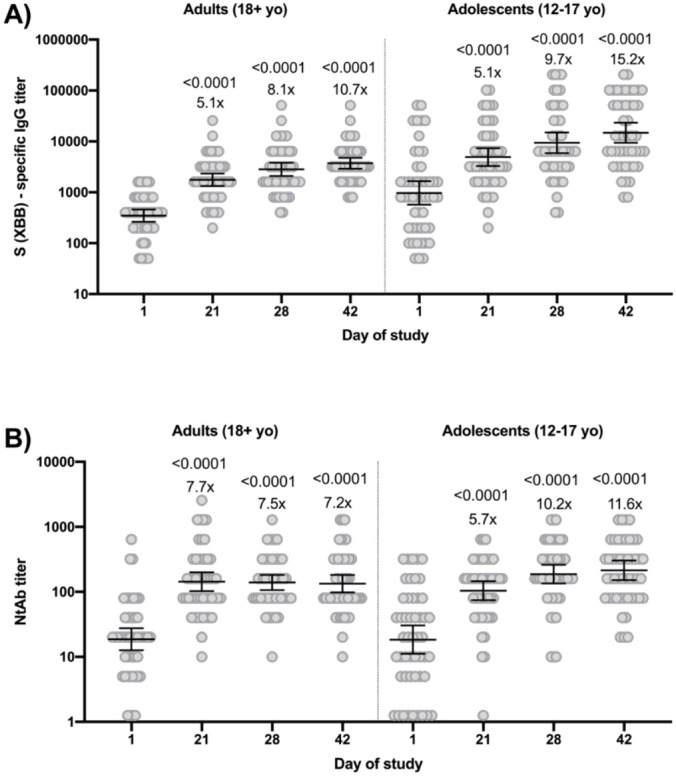
Antigen-specific IgG titers to the SARS-CoV-2 virus S glycoprotein (Omicron XBB) **(A)** and NtAb titers to SARS-CoV-2 XBB.1.5 variant **(B)** in participants’ blood sera on days 1 (before vaccination), 21, 28, and 42 of the study. The figure shows IgG levels, geometric mean, and 95% confidence interval. The figure also shows the significance levels p when comparing IgG levels on days 21, 28, and 42 with day 1 (Wilcoxon test) and fold increase in comparison with day 1.

Analysis of antigen-specific IgG showed a reliable increase in IgG level after vaccination (**Figure 1A**). Thus, a significant increase in the XBB.1 S-specific IgG level was detected in participants: on day 21 of the study, the increase was 5.1 folds, on day 28 - 8.1 and 9.7 folds, on day 42 - 10.7 and 15.2 folds in adults and adolescents, respectively.

Analysis of the increase in the antigen-specific IgG level on day 28 of the study showed that, in general, the increase was detected in 100% participants’ sera, while the level of seroconversion (an increase in antibody titer by 4 or more times) was 90% for adults and 83.7% for adolescents, on day 42 of the study the level of seroconversion was 100% for adults and 87.5% for adolescents.

Analysis of NtAb in participants’ blood sera showed a significant increase in the NtAb level (**Figure 1B**). Thus, NtAb against XBB.1.5 increased ~7 folds in adults and ~11 folds in adolescents after vaccination.

**Figure 2:**
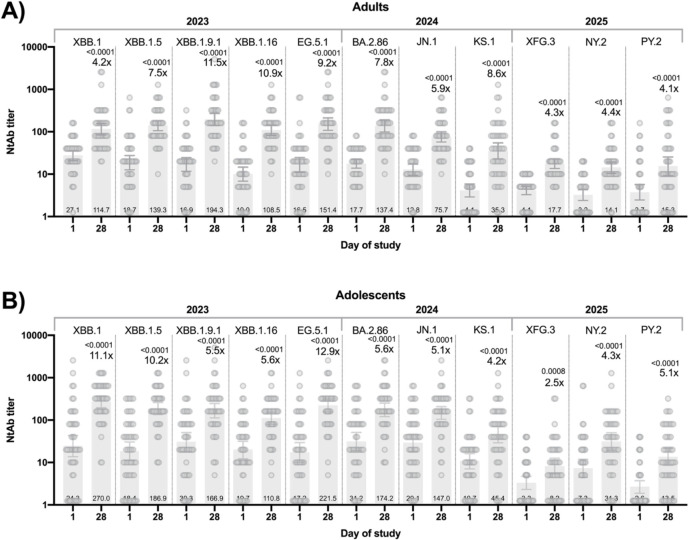
NtAb titers to different SARS-CoV-2 variants (circulated in 2023-2025) in participants’ blood sera **(A)**. adults, **(B)**. adolescents) on days 1 (before vaccination) and 28 of the study. The figure shows NtAb levels, geometric mean, and 95% confidence interval. The figure also shows significance p-levels when comparing NtAb levels with day 1 (Wilcoxon test) and fold increase in comparison with day 1.

During the studies the spectrum of circulating SARS-CoV-2 variants has changed and we, additionally, studied NtAb response to different circulating variants (from XBB.1 in 2023 to PY.2 in 2025, **Figure 2**). Overall, we detected a significant increase in NtAb for all studied variants, which indicates the effectiveness of the Sputnik V XBB in inducing broad-specific NtAb. The strongest increase was detected for variants circulating in 2023-2024 (from XBB.1 to KS.1) (**Figure 2**).

**Figure 3:**
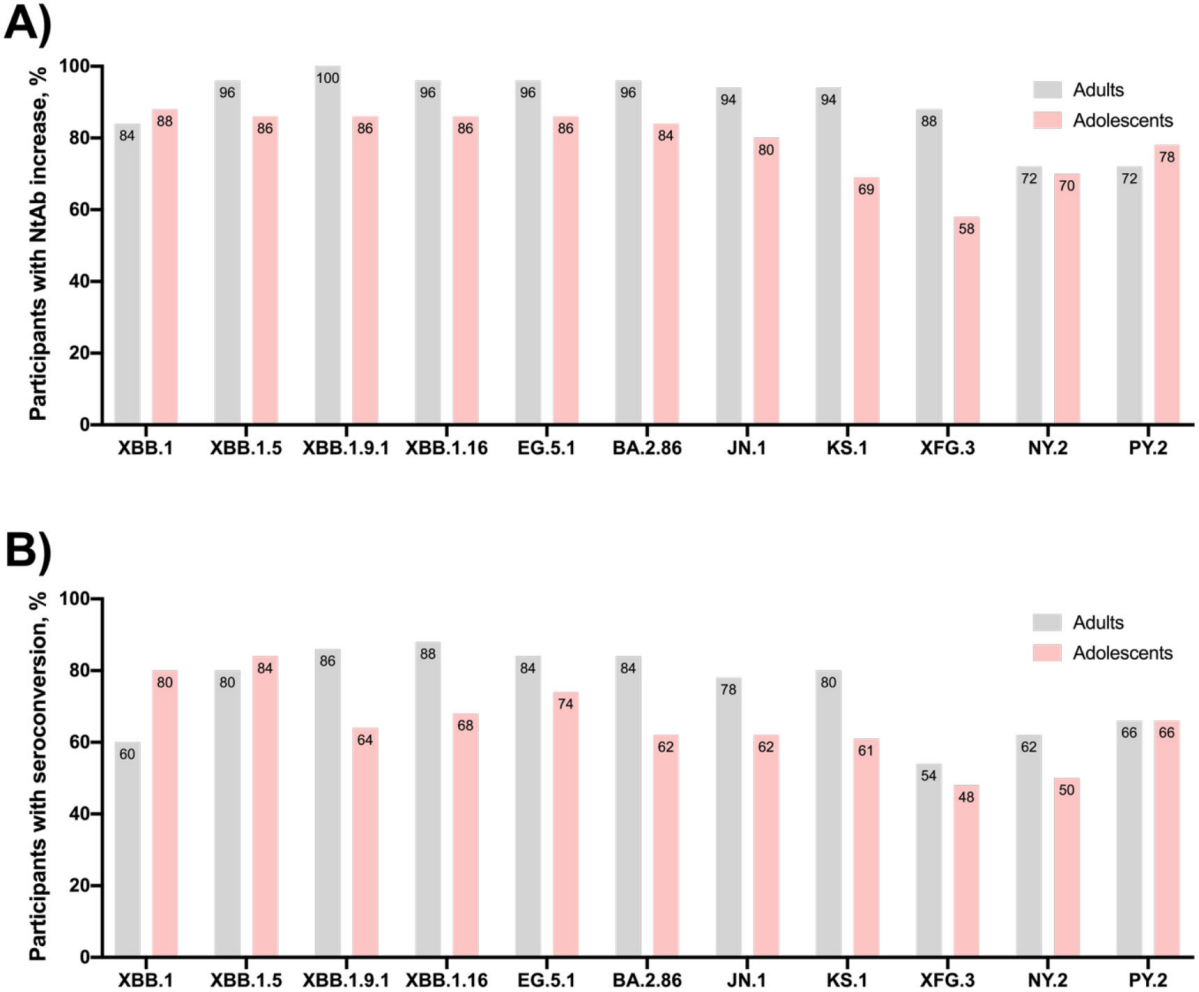
Participants achieving **(A)** increase in NtAb response and **(B)** NtAb seroconversion (an increase in antibody titer by 4 or more times) 28 days after first dose of Sputnik V XBB (grey – adults, pink – adolescents).

Four weeks from the first dose of Sputnik V XBB vaccine, >90% adults and >80% adolescents showed an increase in neutralizing antibodies to different SARS-CoV-2 variants circulated in 2023-2024 (XBBs, EG.5.1, BA.2.85, JN.1, KS.1) (**Figure 3**). Seroconversion analysis showed that, in general, the seroconversion rate in adults was higher than in adolescents and amounted to 78-88% for the variants circulated in 2023–2024 in adults and 62-84% in adolescents. Overall, when analyzing a 4-fold NtAb increase to any of the SARS-CoV-2 variants, we detected seroconversion in 96% of adults and 94% of adolescents. Seroconversion for variants circulating in 2025 (XFG.3, NY.2, PY.2) in adults and adolescents has decreased to 50-60%.

## 4 Discussion

The introduction of vaccines into clinical practice in different countries has significantly reduced the incidence and severity of COVID-19 among vaccinated people. However, the SARS-CoV-2 virus has been actively evolving since it entered the human population. The spread of new variants of the SARS-CoV-2 virus has led to a decrease in the epidemiological effectiveness of vaccines around the world. With regard to the Alpha variant, the effectiveness in protecting against the disease was more than 86%, with regard to the Beta, Gamma and Delta variants - more than 70%, and with regard to the first sublineages of the Omicron variant, the effectiveness decreased to 23%. It is worth noting that the introduction of booster doses of vaccines made it possible to increase the effectiveness of vaccination against the Omicron variant to 57%. Despite the decrease in effectiveness against morbidity, protection against severe disease and COVID-19-associated deaths remained high during the period of spread of all variants (19, 20).

Given the constant shift in circulating SARS-CoV-2 variants, the declining effectiveness of current vaccines against new variants, and the persistently high incidence of COVID-19, continuous monitoring of vaccine efficacy against new variants is essential. The Gamaleya NRCEM since 2020 conducts monitoring in two branches: studies of the neutralizing activity of vaccinated volunteers’ blood serum and the protective activity of the vaccine in animals (18, 21–24). If a decrease in efficacy is detected, the antigen composition of the vaccines must be updated. These studies are in line with WHO studies, based on the results of which, starting in 2022, WHO issue recommendations on changing the antigen composition of vaccines (25).

The results of laboratory monitoring of the Sputnik V vaccine showed a critical decrease in efficacy against the ХВВ variant: the decrease in the NtAb level in the vaccinated individuals’ blood serum was more than 40 times, and studies of the efficacy of the vaccine with the initial composition in animal model demonstrated that the viral load in the lungs was reduced by only 8 folds. Due to the decrease in efficacy, it was decided to update the antigen composition of the vaccine against the circulating XBB variant. Sputnik V XBB preclinical studies confirmed its high efficacy in animals, which allowed us to move on to clinical trials of a Sputnik V XBB.

During the safety study in adults (18+ yo), 96 AEs were registered, which developed in 35 subjects (70%) after the vaccine administration. All adverse events in adults were of the 1st (mild) severity and were short-term. Most of the AEs (91.7%) in adults resolved on their own without any medication or other therapy. During the safety study in adolescents (12–17 yo), 62 AEs were registered, which developed in 38 subjects (76%) after the vaccine administration. All adverse events in adolescents were of 1st (mild) and 2nd (moderate) severity, and were short-term. Most AEs (87%) in adolescents resolved spontaneously without drug therapy. Adverse events associated with the vaccine administration were mostly local or systemic reactions to the drug administration, typical for the use of immunobiological drugs. No serious AEs were registered during whole study period. The analysis of safety data allowed to conclude that the Sputnik V XBB has a favorable safety profile in adults and adolescents.

The identified AEs of the Sputnik V XBB vaccine are comparable with the AEs identified in the open-label Phase 2/3 Trial XBB.1.5-Adapted BNT162b2 COVID-19 Vaccine in adults (172 volunteers). For example, pain at the injection site (the most common AE for vaccines) was observed in 54% and 76% of participants vaccinated with Sputnik V XBB and BNT162b2 XBB.1.5, respectively. Musculoskeletal pain – 8% and 22%, respectively. Headaches were experienced by 4% and 44%, respectively (26).

During the immunogenicity study, it was shown that the Sputnik V XBB administration led to the induction of a significant increase in neutralizing antibodies against circulated SARS-CoV-2 variants (XBB.1.5, XBB.1.9, XBB.1.16, EG.5.1, BA.2.86, JN.1, KS.1, XFG.3, NY.2, PY.2). The immunogenicity data are also comparable to the most widely used vaccine BNT162b2 XBB.1.5. The increase in the level of neutralizing antibodies in the sera of vaccinated with BNT162b2 XBB.1.5 participants one month after vaccine administration was 5.9, 6.9 and 3.7 for SARS-CoV-2 XBB.1.5, EG.5.1 and BA.2.86, respectively (26). As for the Sputnik V XBB vaccine, the increase was 7.5, 9.2 and 7.8, respectively. Moreover, percent of 18+ participants with seroresponse of NtAb to XBB.1.5, EG.5.1 and BA.2.86 was 80%, 84% and 84%, respectively, for Sputnik V XBB vaccine, while study of BNT162b2 XBB.1.5 (n = 37) showed 68% 73% and 60% seroresponse, respectively (26).

The WHO COVID-19 vaccine composition recommendation for 2024–2025 was to use the JN.1 sublineage as the antigen (27). In our studies, we did not detect a critical decrease in the efficacy of the Sputnik V ХBB vaccine against the JN.1 sublineage, so there was no need to change the antigen composition. Many other developers of well-known vaccines have changed the antigen composition of their vaccines against the JN.1 sublineage, for example, Nuvaxovid™ (Novavax, Inc., Gaithersburg, MD, USA). A phase 3, single-arm, open-label clinical trial of the NVX-CoV2705 vaccine (60 volunteers) showed that administration of the vaccine resulted in a 4.5-fold increase in JN.1-specific NtAbs on day 28 (28). These data are comparable with the results of our studies - the administration of the Sputnik V XBB vaccine leads to an increase in JN.1-specific NtAbs by 5.9 times in adults and 5.1 times in adolescents day 28 from the start of vaccination. Safety studies of the Sputnik V ХBB and NVX-CoV2705 vaccines also showed comparable results. For example, local side effects, such as pain at the injection site, occurred in 54% and 64% of volunteers, respectively. However, systemic reactions were less common with the Sputnik V ХBB vaccine (28).

The clinical studies of Sputnik V XBB had a number of limitations. These studies was open-label non-randomized and did not include a comparatory cohort due to Russian regulatory guidelines for COVID-19 vaccines with updated antigen composition (Resolution of the Government of the Russian Federation of 03.04.2020 N 441, as amended on 30.12.2022). The size of the cohorts was limited, which allowed to report frequent AEs. The observation period of the studies was limited to 90 days for safety assessment and 42 days for immunological studies. Therefore, we were able to investigate only short-term peak antibody responses at 3 weeks after the booster dose. Further studies are needed to define the durability of protection and the time period before the next vaccination. Also, our studies focused on humoral immune responses and did not include cellular immune responses analyses, which limits the knowledge of breadth of cellular immunity, induced by Sputnik V XBB. Considering potential protection of Sputnik V XBB, efficacy data was not reported in this study. However, neutralization titers are considered a strong correlate of COVID-19 vaccine protection and are used by the regulatory organizations as WHO and FDA to approve updated COVID-19 vaccines before gaining real-world efficacy data (29). Therefore, looking back at neutralization data against 11 variants of SARS-CoV-2, Sputnik V XBB showed broad neutralizing response in adults and adolescents: GMT to XBB-lineage variants, circulating in 2023, was not less than 108.5 in adults and 110.8 in adolescents. In both cohorts, neutralization titers to SARS-CoV-2 variants, circulated in 2024 and 2025, predictably showed a decline. However still, at 28 days after vaccination with Sputnik V XBB, neutralizing antibodies to SARS-CoV-2 variants, circulated in 2025, grew up to 4.4-fold in adults and 5.1-fold in adolescents. These results contribute to the knowledge of prospective efficacy of currently available COVID-19 vaccines. The results of the clinical trial demonstrated a favorable safety profile and a high level of immunogenicity of Sputnik V XBB in adults and adolescents, which made it possible to make changes to the drug’s registration dossier. The Sputnik V XBB has been introduced into civil circulation in the beginning of 2024 and used to vaccinate the population as part of the preventive vaccination calendar for epidemiological indications (Order of the Ministry of Health of the Russian Federation dated 12.12.2023 No. 677n).

In 2025, laboratory monitoring of the Sputnik V XBB vaccine’s efficacy revealed a 6.5-fold, 8.1-fold, and 7.5-fold decrease in the neutralizing activity of antibodies in the blood serum of vaccinees against the XFG.3, NY.2, and PY.2 variants, respectively. The geometric mean NtAb levels were 17.6, 14.1, and 15.3, respectively. Given the significant decrease in NtAb, a subsequent decrease in the vaccine’s protective efficacy against newly circulating variants can be expected. Despite the fact that the COVID-19 pandemic has ended, the pathogen continues to circulate in the human population, with new variants of the SARS-CoV-2 virus emerging, causing increases in morbidity among the immune and non-immune population. It is important to continue monitoring the effectiveness of the vaccines used to promptly change the antigen composition and introduce effective drugs into circulation to protect the population.

## Data Availability

The original contributions presented in the study are included in the article/**Supplementary Material**. Further inquiries can be directed to the corresponding author.

## Supplementary material

Supplementary Table 1 Inclusion, non-inclusion and exclusion criteria of the Clinical trial in adults.

Supplementary Table 2 Inclusion, non-inclusion and exclusion criteria of the Clinical trial in adolescents.
