# Supplementary material for: Sputnik V update: safety and neutralizing antibodies in healthy adults and adolescents
Source: Front Immunol. 2026 Mar 26;17:1736771. doi: 10.3389/fimmu.2026.1736771 (PMC13062787; doi:10.3389/fimmu.2026.1736771)
Supplement: Supplementary Table 1 [file DataSheet1.docx]

**Supplementary Table 1: Inclusion, non-inclusion and exclusion criteria of the Clinical trial in adults**

| **Inclusion criteria** | Partiсipants was included in the study if all of the following inclusion criteria were met:  1. Written informed consent of the subject to participate in the study  2. Adult male and female participants over 18 years of age  3. Negative COVID-19 test result, determined by the PCR assay or rapid test before the vaccine administration  4. Consent to use effective contraception during the entire period of participation in the study  5. Negative pregnancy test based on the results of a urine test at the screening visit (for women with preserved reproductive potential)  6. Negative test for the presence of narcotics and psychostimulants in urine at the screening visit  7. Negative test for alcohol content at the screening visit  8. Any vaccination no earlier than the last 30 days before inclusion in the study  9. No contraindications to vaccination*  10. Absence of acute infectious and/or respiratory diseases for at least 14 days prior to inclusion in the study  * If the subject has contraindications to vaccination, determined on the basis of the “Methodological recommendations for the identification, investigation and prevention of adverse events after immunization” (approved by the Ministry of Health of Russia on 12.04.2019), vaccination may be postponed for a period determined by the specified document |
| --- | --- |
| **Non-inclusion criteria** | Participants was not included in the study if at least one of the following non-inclusion criteria was met:  1. Lack of signed informed consent  2. Therapy with steroids (except hormonal contraceptives and/or ongoing hormone replacement therapy) and/or immunoglobulins or other blood products completed less than 30 days prior to inclusion in the study  3. Therapy with any immunosuppressive drugs completed less than 3 months prior to inclusion in the study  4. Female subjects during pregnancy or lactation  5. Acute coronary syndrome or stroke suffered less than one year prior to inclusion in the study  6. Tuberculosis, chronic systemic infections according to the anamnesis  7. Complicated allergic history (history of anaphylactic shock, Quincke's edema, polymorphic exudative eczema, serum sickness), hypersensitivity or allergic reactions to the administration of immunobiological drugs, known allergic reactions to drug components, exacerbation of allergic diseases on the day of inclusion in the study  8. History of neoplasms (ICD codes C00-D09)  9. History of splenectomy  10. History within 6 months prior to inclusion in the study: neutropenia (decrease in absolute neutrophil count less than 1000/mm^3^), agranulocytosis, significant blood loss, severe anemia (hemoglobin less than 80 g/l), immunodeficiency  11. Subjects with a history of active human immunodeficiency virus disease, syphilis, hepatitis B and C  12. Anorexia, protein deficiency of any origin  13. History of alcoholism and drug addiction  14. Subject's participation in any other interventional clinical trial (except rescreening in the current trial) in the last 90 days  15. Extensive tattoos at the injection sites (deltoid muscle area) that do not allow assessment of the local reaction to the vaccine administration  16. Any other condition of the study subject that, in the opinion of the investigator, may prevent completion of the study in accordance with the protocol  17. Vaccination against COVID-19 or a history of coronavirus infection COVID-19 less than 6 months before screening  18. Multiple administration of Sputnik V, Sputnik Light or multiple administration of any other COVID-19 vaccine for more than three injections (Sputnik V vaccination plus Sputnik Light revaccination)  19. Inability to read Russian; inability or unwillingness to understand the essence of the study  20. Any other conditions that limit the validity of obtaining informed consent or may affect the ability of the participants to participate in the study  21. Research center staff (principal investigator and members of the research team) directly involved in the study and their family members. |
| **Exclusion criteria** | Participants stopped undergoing the study procedures and remain under observation until the completion of the study in the following cases:  1. Participants refuses to continue participating in the study (withdrawal of informed consent)  2. Participants fails to comply with the rules of participation in the study  3. Occurrence of reasons/occurrence of situations during the study that threaten the safety of the participant  4. Participants selected for participation in the study with violation of the inclusion/non-inclusion criteria  5. Vaccination with any vaccine (except for the one studied in this study) for the prevention of COVID-19 both in clinical trials and in civil circulation during the study  6. Occurrence of other reasons during the study that prevent the study from being conducted according to the protocol.  In the event of detection of SAEs or the onset of pregnancy, participants continue to be observed. After identification of COVID-19 disease, planned visits within the framework of the study are no longer carried out; a specialized unscheduled visit is carried out for cases of SARS-CoV-2 infection; if an in-person visit is not possible, a visit is carried out in the form of a telephone consultation. |

**Supplementary Table 2: Inclusion, non-inclusion and exclusion criteria of the Clinical trial in adolescents**

| **Inclusion criteria** | Partisipants was included in the study if all of the following inclusion criteria were met:   1. Written informed consent from the study subject and the subject's parents/adoptive parents to participate in the study; 2. Young men and women aged 12-17 inclusive; 3. A negative COVID-19 test result, determined by PCR or a rapid test before vaccination; 4. No history of COVID-19 within 6 months prior to screening; 5. No contact of the study subject with anyone infected with COVID-19 for at least 14 days prior to study inclusion (according to the study participant or the subject's parents/adoptive parents); 6. Consent to use effective contraception throughout the entire study period; 7. A negative urine pregnancy test at the screening visit (for all young women participating in the study); 8. A negative urine test for narcotics and psychostimulants at the screening visit; 9. Negative alcohol test at the screening visit; 10. No history of severe post-vaccination reactions or post-vaccination complications following the use of immunobiological drugs; 11. No history of acute infectious and/or respiratory diseases for at least 14 days prior to study inclusion. |
| --- | --- |
| **Non-inclusion criteria** | Participants was not included in the study if at least one of the following non-inclusion criteria was met:   1. Any vaccination/immunization administered within 30 days prior to study inclusion. 2. Steroid therapy (except hormonal contraceptives) and/or immunoglobulins or other blood products not completed within 30 days prior to study inclusion; 3. Immunosuppressive therapy and systemic corticosteroid therapy completed less than 3 months prior to study inclusion. 4. Acute coronary syndrome or stroke less than one year prior to study inclusion. 5. Any immunodeficiency (e.g., hereditary immunodeficiency, acquired immunodeficiency syndrome [AIDS], etc.). 6. Infectious diseases:   − history of HIV, hepatitis, active syphilis at the time of screening;  − tuberculosis;  − Active infection (except onychomycosis) or any significant episode of infection requiring treatment with intravenous antibiotics within 4 weeks prior to screening or oral antibiotics within 2 weeks prior to screening;  − History of a serious recurrent or chronic infection not listed above.   1. Major surgery within 4 weeks prior to screening. 2. History of chronic autoimmune diseases or systemic collagenoses requiring immunosuppressive therapy. 3. Volunteers who have undergone an organ transplant, including bone marrow or peripheral blood stem cell transplantation, and are receiving immunosuppressive therapy. 4. History of splenectomy. 5. Volunteers with a previous or concomitant history of neoplasms. 6. Complicated allergic history (history of anaphylactic shock, angioedema, and other life-threatening conditions), hypersensitivity or allergic reactions to immunobiological drugs, known allergic reactions to any component of the vaccine or a vaccine containing similar components, exacerbation of allergic diseases on the day of inclusion in the study. 7. Neutropenia (decrease in absolute neutrophil count to less than 1000 cells/mm3), agranulocytosis, significant blood loss, severe anemia (hemoglobin concentration less than 80 g/L), thrombocytopenia (decrease in absolute platelet count to less than 50,000 cells/mm3). 8. Anorexia, protein deficiency of any origin. 9. Volunteers with a BMI value in the range of -2 SD to +2 SD for age. 10. Extensive tattoos at the injection sites (deltoid muscle area) that prevent assessment of the local reaction to the vaccine. 11. Chronic cardiovascular, bronchopulmonary, or neuroendocrine diseases, as well as gastrointestinal, liver, kidney, muscle, or connective tissue diseases in the acute or decompensated stage. 12. Mental disorders (registered with a psychiatrist or drug addiction specialist). 13. Diseases that, in the opinion of the study physician, put the participant's health at risk if they participate in the study or potentially complicate the interpretation of the examination results. 14. Family members of research center personnel directly involved in the study, etc. 15. Participation in other clinical trials and use of other investigational drugs within 90 days prior to screening. 16. Planned vaccination against COVID-19 with any vaccine, either as part of other studies or through civilian use. 17. COVID-19 vaccination or history of COVID-19 infection less than 6 months prior to screening. 18. Female subjects during pregnancy or breastfeeding. 19. Inability to read Russian; inability or unwillingness to understand the study. Any other conditions that limit the validity of informed consent or may affect the volunteer's ability to participate in the study. |
| **Exclusion criteria** | Participants stopped undergoing the study procedures and remain under observation until the completion of the study in the following cases:   1. Volunteer's refusal to continue participating in the study (withdrawal of informed consent). 2. Volunteer's failure to comply with the study rules. 3. The emergence of situations during the study that threaten the volunteer's safety. 4. Volunteers selected for participation in the study who violated the inclusion/exclusion criteria. 5. Vaccination with any vaccine (other than the one being studied in this study) for the prevention of coronavirus infection (COVID-19), either in clinical trials or through civilian use during the study. 6. A positive pregnancy test in female subjects. 7. The emergence of other reasons during the study that prevent the study from being conducted according to the protocol.   In the event of the development of SAEs or the onset of pregnancy, volunteers were excluded from the vaccination program; however, they continued to be monitored in accordance with the rules described in the study protocol. In the event of SARS-CoV-2 infection (laboratory and/or instrumental confirmation), the volunteer is excluded from the vaccination program, but not from the study. After the disease is identified, standard study visits are no longer conducted, but at least one specialized unscheduled visit is conducted for cases of SARS-CoV-2 infection. |
